# The role of Mean Platelet Volume/platelet count Ratio and Neutrophil to Lymphocyte Ratio on the risk of Febrile Seizure

**DOI:** 10.1038/s41598-018-33373-3

**Published:** 2018-10-11

**Authors:** Zhigang Liu, Xiangxin Li, Meipin Zhang, Xiaofei Huang, Jun Bai, Zhiwei Pan, Xiuxian Lin, Dongni Yu, Huaping Zeng, Ruiping Wan, Xingguang Ye

**Affiliations:** 1 Department of pediatrics, Southern Medical University Affiliated Maternal & Child Health Hospital of Foshan, 11 Renminxi Road, Foshan, Guangdong 528000 China; 2 Department of Clinical Laboratory, Southern Medical University Affiliated Maternal & Child Health Hospital of Foshan, 11 Renminxi Road, Foshan, Guangdong 528000 China

## Abstract

Systemic inflammatory response has been implicated as a contributor to the onset of febrile seizures (FS). The four novel indices of the inflammatory response such as, neutrophil-to-lymphocyte ratio (NLR), mean platelet volume (MPV), platelet count (PLT) ratio and red blood cell distribution width (RDW) have been investigated in FS susceptibility and FS types (simple febrile seizure and complex febrile seizure). However, the potential role of these inflammatory markers and MPV**/**PLT ratio (MPR) in Chinese children with FS has yet to be fully determined. This study investigated the relevance of NLR, MPV, PLT, MPR and RDW in febrile children with and without seizures. 249 children with FS and 249 age matched controls were included in this study. NLR and MPR were calculated from complete blood cell counts prior to therapy. Differences in age, gender and these inflammatory markers between the FS group and the control group were evaluated using the chi-square test, *t-*test or logistic regression analysis. Receiver Operating Characteristic (ROC) curve was used to determine the optimal cut-off value of NLR and MPR for FS risk. Interactions between NLR and MPR on the additive scale were calculated by using the relative excess risk due to interaction (RERI), the proportion attributable to interaction (AP), and the synergy index (S). It has been shown that the elevated NLR and MPR levels were associated with increased risk of FS. The optimal cut-off values of NLR and MPR for FS risk were 1.13 and 0.0335 with an area under the curve (AUC) of 0.768 and 0.689, respectively. Additionally, a significant synergistic interaction between NLR and MPR was found on an additive scale. The mean levels of MPV were lower and NLR levels were higher in complex febrile seizure (CFS) than simple febrile seizure (SFS), and the differences were statistically significant. ROC analysis showed that the optimal cut-off value for NLR was 2.549 with 65.9% sensitivity and 57.5% specificity. However, no statistically significant differences were found regarding average values of MPR and RDW between CFS and SFS. In conclusion, elevated NLR and MPR add evidence to the implication of white cells subsets in FS risk, and our results confirmed that NLR is an independent, albeit limited, predictor in differentiating between CFS and SFS. Moreover, NLR and MPR may have a synergistic effect that can influence the occurrence of FS.

## Introduction

Febrile seizures (FS), also known as febrile convulsions, affecting approximately 2–5% of children aged six months to five years, are typically divided into two types, i.e., simple febrile seizures (SFS) and complex febrile seizures (CFS)^1,2^. FS are associated with rapidly rising fever without evidence of intracranial infection, metabolic disturbance, or history of afebrile seizures^1,2^. Although fever is a common symptom in children, only some children with fever experience FS, and it is not well understood how fever generates FS. Fever is induced by pro-inflammatory cytokines such as interleukin (IL)-1β, IL-6, and tumor necrosis factor (TNF)-α during infections^3^. To date, many studies have suggested that inflammation, which is intrinsic to the fever response, is involved in the generation of FS^3–9^. These studies suggested that inflammatory cytokines, especially IL-1β, IL-6 and TNF-α can play important role in the generation of FS. Although inflammatory cytokines are useful biomarkers, their increased cost and limited availability are drawbacks.

Peripheral blood neutrophil-to-lymphocyte ratio (NLR), mean platelet volume (MPV) and red blood cell distribution width (RDW) are three novel indices for inflammation. NLR is an indicator of systemic inflammatory response and has been implicated in the pathogenesis of a number of diseases, especially in cardiac diseases and malignancies^10–14^. MPV is a machine-calculated measurement of the average size of platelets. It reflects the platelet size and the rate of platelet production in bone marrow, and may be used as an indicator of platelet activation and severity of inflammation^15–17^. Activated platelets release a large number of substances that are crucial mediators of inflammation^18^. Moreover, several studies have demonstrated an inverse relationship between MPV and platelet counts in critically ill patients^19,20^. Emerging evidence suggests that the combination of platelet count and MPV may be more clinically significant than platelet count or MPV alone^19,21–23^. In addition, the interaction between neutrophils and activated platelets also occurs during the inflammatory response in the blood^24^. RDW has been reported to be positively correlated with inflammatory markers such as the erythrocyte sedimentation rate (ESR), C-reactive protein (CRP) levels and inflammatory cytokines in various diseases^25,26^. Therefore, as widely-used, low-cost host inflammatory response markers, NLR, MPV, PLT and RDW have gained increasing attention as independent predictors associated with FS susceptibility and FS types^27–33^.

Clinical studies have attempted to address the relationship in the blood of febrile children with and without seizures by comparing levels of MPV, NLR, PLT and RDW, but these studies have reported inconsistent results. And it is unknown whether these inflammatory markers and MPR are associated with FS susceptibility and FS types in Chinese children. It is even unknown whether the interaction between NLR and MPR can affect FS susceptibility. Here, to address these questions, we analyzed the association between these inflammatory factors and FS in Chinese children.

## Material and Methods

### Patients and Methods

The study was approved by the Ethics Committee of Southern Medical University Affiliated Maternal & Child Health Hospital, Foshan (China). All experiments and methods were performed in accordance with the relevant guidelines and regulations. A retrospective study was conducted in the department of pediatrics, Southern Medical University Affiliated Maternal & Child Health Hospital, Foshan (China). The hospital admits approximately 440,000 children annually. At recruitment, informed consent was obtained from parents of each child.

A total of 867 children (aged 5 months to 6 years) presented to the department of pediatrics or emergency department because of fever with seizures between January 1, 2015, and December 31, 2017. Of them, 416 (48.0%) patients who not had blood routine test (BRT) obtained within 2 hours after FS were excluded. To control the potential confounding factors and ensure accurate identification of all eligible children, subjects with conditions known or suspected to cause seizures without fever were systematically excluded using sequential, stringent exclusion criteria from the final analysis. Exclusion criteria included prematurity (age at birth <37 weeks and age under one year); a history of FS or epilepsy; a family history of genetic and neurological diseases; subsequent epilepsy after FS; abnormalities of amino acids, organic acid analyses and brain magnetic resonance imaging; electrolyte disorders; static encephalopathy; hydrocephalus; meningitis; viral central nervous system (CNS) infection; previous intracranial infection; mental retardation; stroke; demyelinating disease or ventricular shunt. Patients with acute or chronic systemic disease, eg, cancer and hematologic or rheumatologic disorders, were also excluded. Of these, 133 (15.3%) met sequential exclusion criteria. 69(8.0%) patients without informed consent and were also excluded. The remaining 249 (28.7%) subjects and 249 age matched healthy controls were included in the final analysis. Patients were divided into two groups: one group consisting of 249 children (82 children with complex febrile seizure and 167 children with simple febrile seizure) with febrile seizure and a control group of 249 children with fever of unknown etiology without seizures.

A diagnosis of FS was determined according to the International Classification of Diseases, Ninth Revision (ICD-9) codes (ICD-9 780.31, 780.32).

### Laboratory Analysis

White blood cell count (WBC), red blood cell count (RBC), hemoglobin (Hb), hematocrit (Hct), mean corpuscular volume (MCV), red blood cell distribution width (RDW), mean platelet volume (MPV), platelet count (PLT), monocytes, neutrophil and lymphocyte counts and percentages, were measured from peripheral venous blood samples collected in EDTA tubes during admission. The blood samples were taken and measured 2 h after FS. NLR was calculated by dividing the absolute neutrophil count by the absolute lymphocyte count. MPR was calculated by dividing the MPV by the platelet count.

#### Statistical analysis

Statistical analysis was performed by using the SPSS Statistics 19.0 for Windows (SPSS Inc., Chicago, IL, USA) program. Parametric data (quantitative) was expressed as numbers and percentages; qualitative data were expressed as a mean ± standard deviation. Independent samples t-test was used for comparison of independent data. The Mann-Whitney U-test was used for evaluation of parametric data without binomial distribution. When appropriate, *X*^2^-test was used for the comparison of categorical data. Receiver operating characteristic (ROC) curve analysis was used for calculating the optimal cut-off values, sensitivity, and specificity of NLR and MPR. The optimal cut-off value was determined by Youden’s index. The NLR and MPR were dichotomized according to their optimal cut-off values of ROC curve. In addition, logistic regression was used to calculate the odds ratios (ORs) and their relative 95% confidence intervals (CIs) for risk estimation. Significant associations were defined as *P* < 0.05.

Then, we explored the additive interaction between MPR and NLR according to the following strategy^34^. Among cases and controls, a binary classification was used both for MPR (High vs. Low) and NLR (High vs. Low). The risk for FS for a given MPR and NLR was expressed by the index (i) and (j), where the first index (i) indicated the MPR status coded as 0 for Low subjects and 1 for High subjects, and the second index (j) indicated the NLR, which was coded as 0 for Low subjects and 1 for High subjects. Subjects who were Low MPR and Low NLR were considered as the reference group, and their FS risk was coded as OR_00_ = 1. The relative ORs were obtained by logistic regression. The CIs were calculated by the regression coefficients and corresponding covariance matrix^35^. Deviation from an additive model was calculated as the relative excess risk due to interaction (RERI), the proportion attributable due to interaction (AP), and the synergy index (S). Biological interactions in the regression models were tested as departure from additivity. Based on the adjusted ORs obtained in the logistic regression models, an Excel spreadsheet (www.epinet.se) was used to calculate RERI, AP and S on an additive scale and its corresponding CIs^35^. RERI and AP values (95% CI) that does not cross 0, and S value (95% CI) that does not cross 0 indicates a biological interaction^36^. In addition, RERI > 0 or AP > 0 or S > 1 indicates a positive interaction or more than additivity and RERI < 0 or AP < 0 or S < 1 indicates a negative interaction or less than additivity^37,38^.

## Results

### Characteristics of the study population

A total of 498 patients were included in the study. The mean age of the FS group (n = 249, 69.1% males) was 27.3 ± 14.8 months. The FS group was further divided into two groups such as the SFS group (n = 167, 66.5% males) and the CFS group (n = 82, 74.4% males). The mean age of the SFS group and the CFS group were 26.9 ± 13.5 months and 28.1 ± 17.3 months, respectively. The mean age of the control group (n = 249, 58.2% males) was 25.0 ± 16.5 months. As shown in Table 1, there were no significant differences between the groups in term of age (*P* > *0.05*). However, the frequency of FS was significantly higher in males than controls (*P* < *0.05*).

**Table 1:** Age and gender of the patients.

Variables	Control group (n = 249)	FS group (n = 249)	SFS group (n = 167)	CFS group (n = 82)	*P value*	
					FS *VS*. Control	CFS *VS*. SFS
Age(months)	25.0 ± 16.5	27.3 ± 14.8	26.9 ± 13.5	28.1 ± 17.3	0.099	0.566
Gender						
Male	145 (58.2%)	172 (69.1%)	111 (66.5%)	61 (74.4%)	**0.012**	0.204
Female	104 (41.8%)	77 (30.9%)	56 (33.5%)	21 (25.6%)		

Values are expressed as mean ± standard deviation. FS: febrile seizure. SFS: simple febrile seizure, CFS: complex febrile seizure.

### Association between laboratory test results and febrile seizures

**Figure 1:**
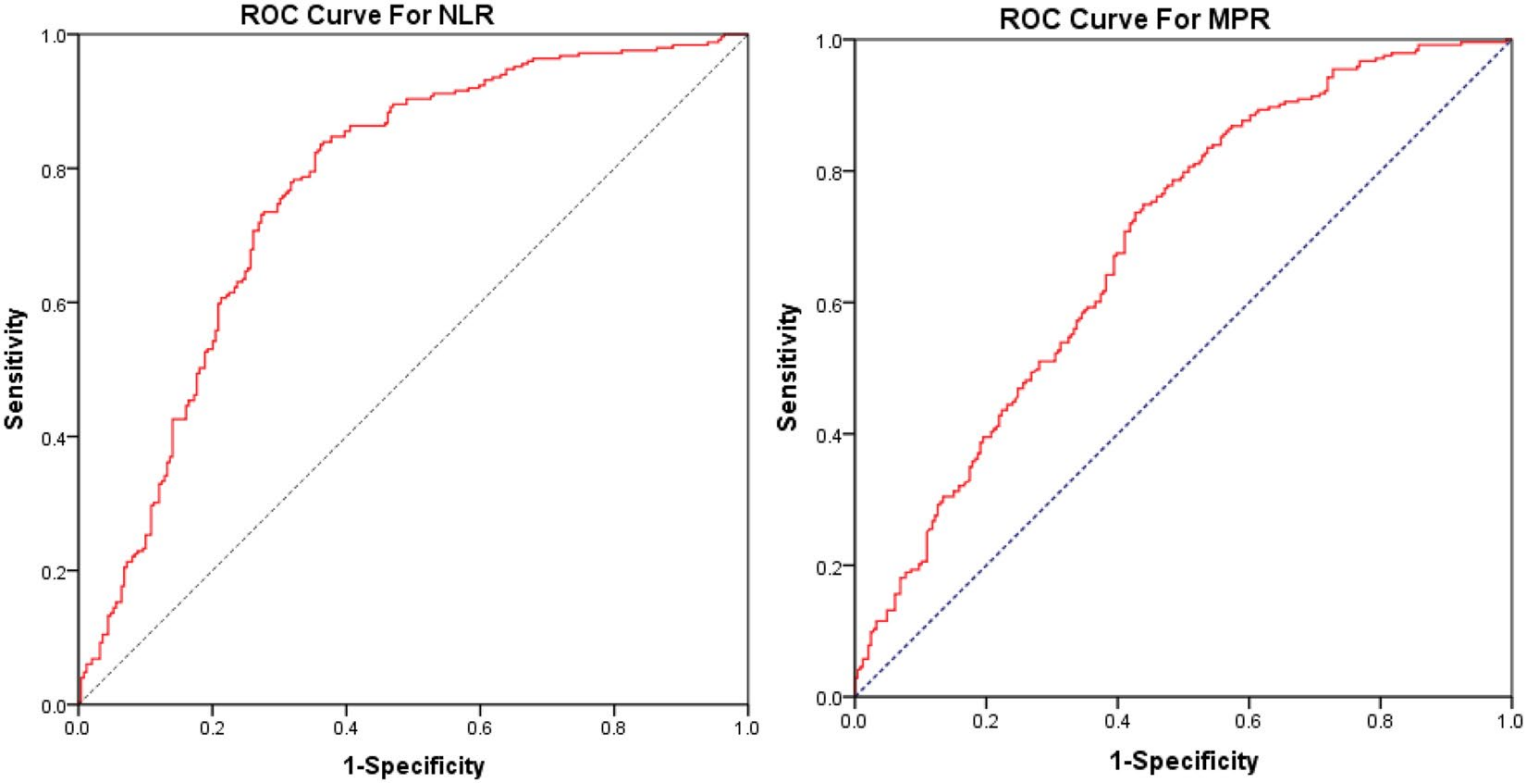
ROC curves of NLR (**A**) and MPR (**B**) for predicting febrile seizure in children with fever.

Then, we assessed the selected laboratory results in febrile children with or without seizures. The results showed that the mean levels of NLR, MPV, MPR, and the number and percentage of neutrophils were significantly higher, while PLT and the number and percentage of lymphocytes were significantly lower in the FS group, compared to the control group (Table 2). The best NLR and MPR cut-off values were ≥1.13 vs. <1.13 and ≥0.0335 vs. <0.0335, respectively, as identified by ROC curve analysis (Fig. 1). Logistic regression analysis revealed that after adjusting for age and gender, NLR ≥ 1.13 was associated with a 10.92-fold increased FS risk compared with the NLR < 1.13 (95% CI: 5.93–13.88, *P* < 0.001); and MPR ≥ 0.0335 was associated with a 3.76-fold increased FS risk compared with the MPR < 0.0335 (95% CI: 2.55–5.53, *P* < *0.001*) (Table 3).

**Table 2:** Laboratory tests performed in children in the FS group and the control group.

Laboratory parameters	Control group (n = 249)	FS group (n = 249)	*P value*
WBC (10^3^/mm^3^)	10.2 ± 3.7	10.6 ± 4.0	0.241
RBC (10^6^/mL)	4.5 ± 0.4	4.5 ± 0.4	0.759
Hb (g/dl)	11.8 ± 1.1	11.8 ± 1.0	0.437
Hct (%)	36.0 ± 3.0	35.6 ± 3.0	0.114
MCV (fl)	79.8 ± 6.1	79.2 ± 6.1	0.253
**MPV (fL)**	**9.6 **±** 0.9**	**9.8 **±** 1.1**	**0.010**
**PLT (10** ^**9**^ **/L)**	**310.6 **±** 112.6**	**236.2 **±** 74.8**	**<0.001**
**MPR**	**0.036 **±** 0.015**	**0.047 **±** 0.020**	**<0.001**
**Neutrophils (%)**	**42.4 **±** 19.7**	**61.1 **±** 15.8**	**<0.001**
**Neutrophils count (x10** ^**3**^ **/mm** ^**3**^ **)**	**4.6 **±** 3.4**	**6.7 **±** 3.5**	**<0.001**
**Lymphocytes (%)**	**46.0 **±** 19.1**	**27.8 **±** 14.3**	**<0.001**
**Lymphocytes count (x10** ^**3**^ **/mm** ^**3**^ **)**	**4.4 **±** 2.3**	**2.8 **±** 1.7**	**<0.001**
**NLR**	**1.6 **±** 1.9**	**3.2 **±** 2.4**	**<0.001**
Monocytes (%)	9.5 ± 3.8	10.1 ± 3.9	0.095
Monocytes count (x10^3^/mm^3^)	0.95 ± 0.52	1.02 ± 0.46	0.142
RDW (%)	13.7 ± 1.4	13.5 ± 1.4	0.096
**CRP (mg/L)**	**14.0 **±** 23**	**9.4 **±** 12**	**0.007**

Values are expressed as mean ± standard deviation. SFS: simple febrile seizure, CFS: complex febrile seizure. CRP: C-reactive protein. Bold text indicates statistical significance.

**Table 3:** Logistic regression analysis of the association of factors (NLR, MPR) and FS.

		Control group		FS group		OR(95% CI)	*P*	OR_adjusted_ (95% CI)^a^	*P* ^a^ *value*
		NO.	%	NO.	%				
NLR	<1.13	158	63.5	40	16.1	1 (Ref)	N/A	1 (Ref)	N/A
	≥1.13	91	36.5	209	83.9	**9.07 (5.93–13.88)**	**<0.001**	**10.92 (5.93–13.88)**	**<0.001**
MPR	<0.0335	141	56.6	63	25.3	1(Ref)	N/A	1(Ref)	N/A
	≥0.0335	108	43.4	186	74.7	**3.85 (2.64–5.64)**	**<0.001**	**3.76 (2.55–5.53)**	**<0.001**

OR, odds ratio; NO, number of subjects; CI, confidence interval; ^a^Adjusted for age and gender; N/A, Not applicable; Bold text indicates statistical significance.

### MPR-NLR interaction for Febrile Seizure susceptibility

Additionally, the interactive effects of MPR and NLR based on an additive scale were also evaluated. According to the RERI, AP and S indexes, a significant synergistic interaction was found between MPR and NLR (AP = 0.57, 95% CI = 0.36–0.77; S = 2.35, 95% CI = 1.43–3.87) (Table 4).

**Table 4:** The interaction of risk estimates between MPR and NLR based on the additive scale.

MPR^b^	NLR^c^	Cases (%)	Controls (%)	OR (95% CI)	*P* ^a^ *value*
−	−	33 (13.3)	66 (26.5)	1	
+	−	7 (2.8)	92 (36.9)	6.69 (2.87–15.58)	**<0.001**
−	+	168 (67.5)	50 (20.1)	18.75 (8.02–43.85)	**<0.001**
+	+	41 (16.5)	41 (16.5)	56.15 (24.21–130.21)	**<0.001**
RERI (95%CI) = 31.71 (−0.41~63.84)					
AP (95%CI) = 0.57 (0.36~0.77)					
S (95%CI) = 2.35 (1.43~3.87)					

^a^Adjusted for age (months) and gender; ^b^MPR (−<0.0335; +≥0.0335); ^c^NLR (−<1.13; +≥1.13); OR, odds ratio; CI, confidence interval; RECI, excess risk due to interaction; AP, attributable proportion due to interaction; S, the synergy index; Bold values are statistically significant.

### Differences in the selected laboratory results between Children with Simple and Complex Febrile Seizures

**Figure 2:**
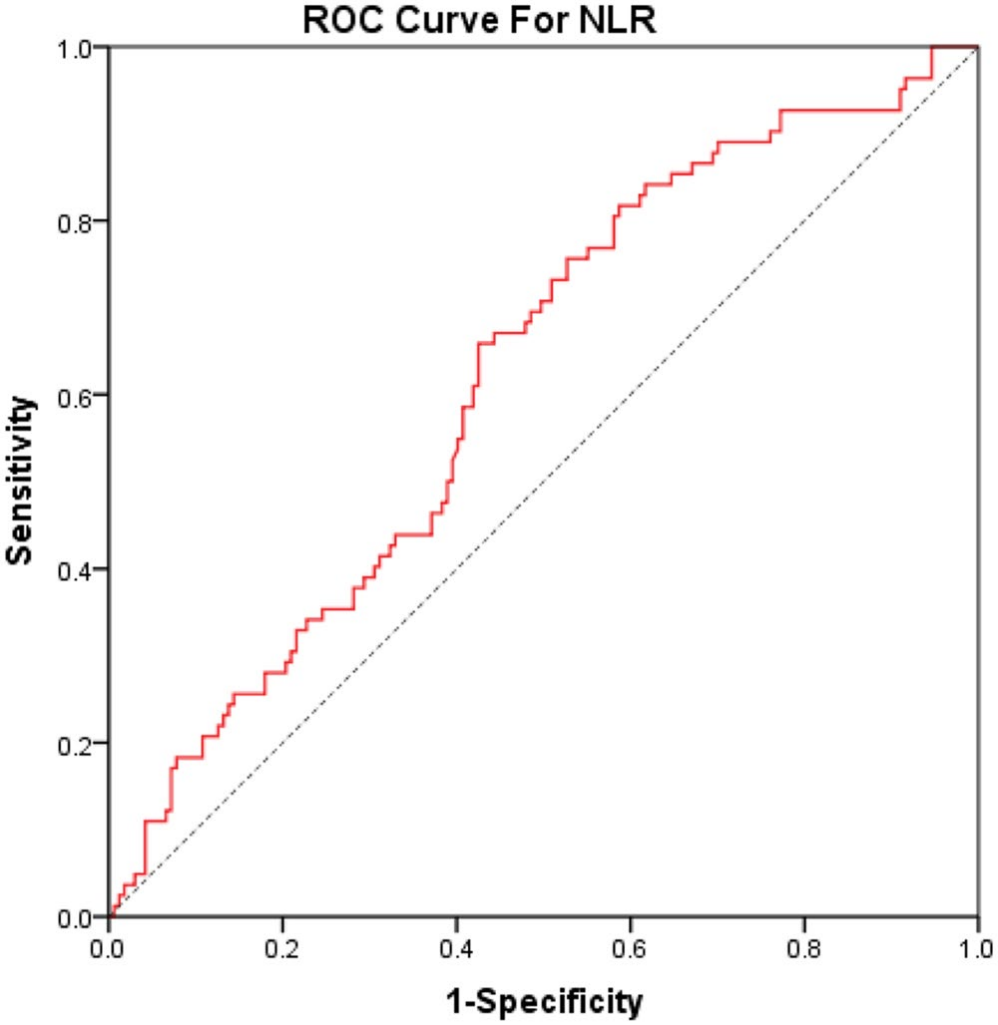
ROC curve analysis of neutrophil lymphocyte ratio (NLR) in differentiating between simple and complex febrile seizures. Area under the curve (AUC, 0.665; confidence interval CI, 0.573–0.756).

Then, the selected laboratory results were also assessed between the SFS group and the CFS group. The results showed that NLR was significantly lower (SFS vs. CFS: 2.9 ± 2.4 vs. 3.7 ± 2.5), while MPV was significantly higher (SFS vs. CFS: 10.0 ± 1.0 vs. 9.4 ± 1.1) in the SFS group, compared with the CFS group (Table 5). However, no statistically significant differences were found regarding average values of MPR and RDW between CFS and SFS. According to ROC curve analysis in differentiating between SFS and CFS (Fig. 2), the optimal cut-off value for NRL was found to be 2.549 (65.9% sensitivity, 57.5% specificity, AUC: 0.620) (Table 6).

**Table 5:** Mean values and standard deviations of MPV, PLT, MPR, NLR, and RDW among SFS and CFS patients.

Variables	SFS group (n = 167)	CFS group (n = 82)	*P value*
**MPV (fL)**	**10.0 **±** 1.0**	**9.4 **±** 1.1**	**<0.001**
PLT (10^9^/L)	233.3 ± 75.2	242.0 ± 74.1	0.392
MPR	0.048 ± 0.019	0.044 ± 0.023	0.175
**NLR**	**2.9 **±** 2.4**	**3.7 **±** 2.5**	**0.014**
RDW (%)	13.5 ± 1.3	13.4 ± 1.5	0.762
CRP	9.4 ± 11	9.5 ± 13	0.936

Values are expressed as mean ± standard deviation. SFS: simple febrile seizure, CFS: complex febrile seizure. CRP: C-reactive protein. Bold text indicates statistical significance.

**Table 6:** ROC curve analysis results of NLR in SFS and CFS patients.

Variables	Cut-off value	FN	TP	TN	FP	Sensitivity	Specificity	AUC, 95% CI
NLR	2.549	28	54	96	71	65.9%	57.5%	0.620 (0.548–0.692)

FN: false negative, TP: true positive, TN: true negative, FP: false positive, AUC: Area Under the Curve.

## Discussion

Although numerous studies have been performed on the identification of factors causing FS in children, the pathophysiology of FS is incompletely understood^39,40^. Accumulating evidence suggests a link between FS and inflammation^5,41–43^. Since FS occur during the course of a high body temperature or a rapidly rising fever in susceptible individuals, various factors associated with fever generation could be involved in the possible causative mechanism of FS. Fever generation involves many cytokines and endogenous mediators, including IL-1β, IL-6, TNF-α, IL-1Ra, and IL-10, in response to exogenous pyrogens. Of these, IL-1β and TNF-α are the important cytokines in the development of FS^41,44^. One of the important role of IL-1β and TNF-α is direct and indirect modulating effects on neurons and neurotoxic neurotransmitters released during excitation or inflammation^45^.

Additionally, inflammation is a mechanism of innate immunity, this process involve the major subtypes of immune system cells, including monocytes, macrophages, neutrophils, lymphocytes, basophils, eosinophils, dendritic cells, and mast cells. Immune system activation is of evident importance in patients with FS^46^. So, this question was raised to us; what is the role of subtypes of immune system cells in FS? Previous studies supporting the hypothesis that associated increased cytokines play an important role in the development of FS indicate that during infections, immune cells such as macrophages, and lymphocytes are stimulated and consequently secrete proinflammatory cytokines such as IL-1β, TNF-α, and IL-6^47–49^. Helminen *et al*.^50^ and Matsuo *et al*.^51^ studied peripheral blood monocytes in children with FS and found that peripheral blood mononuclear cells obtained at the time of FS were observed to produce more IL-1β than peripheral blood mononuclear cells obtained from control subjects. Matsuo *et al*.^51^ also showed that induction of leukocytes by double-stranded RNA resulted in a large-scale production of IL-1β in FS patients as compared to that in controls. IL-1β also stimulates the secretion of cortisol^52–54^. Cortisol induces leukocytosis, neutrophilia, and lymphopenia^55^. Woiciechowsky *et al*.^56^ also reported that intracerebroventricular infusion of IL-1β (but not TNF-α) dramatically increased peripheral neutrophil counts, whereas lymphocytes dropped. Romanowska *et al*.^28^ showed that children with FS had statistically significant higher neutrophils level compared to those with fever without seizures and the number of lymphocytes was lower in children with FS than in children with fever without seizures. In addition, Oguz *et al*.^57^ showed that children with FS had lower blood CD3 and CD4 values than healthy children. Except for the above studies, there is no information on the role of other subtypes of immune system cells in the development of FS, still this question remains unanswered for us, whether subtypes of immune system cells related to the effect of seizure attack during infection? According to my knowledge and literature review there is no document now to have satisfactory answer for this question. Responding to this question, more extensive study including FS group and febrile control group with the values of subtypes of immune system cells and IL-1β should be performed to clarify this point.

In this study, our main objective is to identify peripheral blood markers of FS and for that purpose; we are looking at the profile of white blood cells subsets focusing on monocytes, neutrophils, and lymphocytes. We did not include eosinophils and basophils, because they are granulocytic white blood cells that are rare in humans. The role of eosinophils in immunity remains enigmatic. They have been recognized as crucial players in allergic inflammation, but there is no document now to reveal an association between them and FS. As a result, we found children with FS had statistically significant higher neutrophils level and lower lymphocytes level in children with FS than in children with fever without seizures. And, no significant association was found between monocytes and FS.

NLR is a measure of the proportion of systemic neutrophils and lymphocytes, and may serve as an emerging parameter that reflects systemic inflammation of various diseases. In recent years, NLR has been suggested as one of practical predictor for differentiating FS types^29,31^. In this study, we found that febrile children with FS had statistically significant higher NLR levels compared to those without seizures. Romanowska *et al*.^28^, also found the similar results, which is the only related report in the literature. These findings suggested that elevated NLR is independently and strongly associated with increased risk of FS in Chinese children. Although the mechanism underlying the association is complex and remains to be elucidated, it may be related to increased neutrophil-dependent inflammation, and reduced lymphocyte mediated anti-inflammation response^58,59^. First, neutrophils are specialist cells of the innate immune system that play a major role in host defense through phagocytosis and generation of reactive oxygen species (ROS)^60,61^. Previous studies have demonstrated that there exists a cause and effect relationship between ROS production and epileptic seizures^62–64^. Second, neutrophils are the first cells to migrate into the area of injury as part of the host defense system, and can induce the secretion of several inflammatory cytokines associated with the risk of FS, especially IL-1β and TNF-α play an important role in the pathogenesis of FS^41,65^. The inflammation, thus triggered by these molecules can lead to further inflammation due to cell dysfunction^61,66,67^. Third, recent studies have reported that the voltage-gated sodium channels can comprise a family of nine alpha subunits (referred to as NaV1.1 through NaV1.9), which are variably expressed in leukocytes. A subset of neutrophils recruited to the site of injury has been demonstrated to express NaV1.3 sodium channels^68,69^. Na_V_1.3 channels can recover from inactivation rapidly and sustain high-frequency firing^70^. These channels can be activated during slow ramp depolarizations, and can produce persistent sodium current^71–74^. Finally, a low lymphocyte count specifies that the body’s resistance to fight infection is substantially reduced. Thus, neutrophils and lymphocytes play important role in the process of FS. Elevated NLR is associated with increased risk of FS.

Platelet activation has been reported as a common phenomenon in cardiovascular diseases and several malignancies, and inhibition of platelet activation could ameliorate inflammation^75–78^. MPV and platelet count are two main characteristics to evaluate platelet activation^79^. Elevated MPV is an indicator of larger, more reactive platelets resulting from an increased platelet turnover, and it may be used as an indicator of platelet activation and severity of inflammation^16^. Recent studies have shown that the platelet count was significantly lower^28,30,33^ and MPV was significantly higher^30^ in children with FS compared to febrile children without FS. In the present study, we also found that the MPV value was higher in the FS group; however, the value of platelet count was lower. Therefore, we suspected that there is an association between the FS risk and platelet activation, and MPR (measured by MPV and platelet count) could serve as a marker for FS risk. Our findings demonstrated that MPR may be a reliable predictive marker for FS risk. Although the underlying mechanisms are still to be elucidated, several hypotheses can be proposed. Recent study has suggested that platelet activation maybe a consequence of enhanced bacterial lipopolysaccharide (LPS) circulating levels^80^. Bacteria may activate platelets^81^; thus, the association between FS caused by the bacterial infection and *in vivo* platelet activation is biologically plausible. On the other hand, platelets can store a number of pro-inflammatory (IL-1α, IL-1β, and TGF-β1) and regulatory mediators (serotonin, dopamine, epinephrine, histamine, and GABA) in their granules, and are promptly released at the sites of inflammation or tissue injury during platelet activation^79,82^. Moreover, activated platelets may bind to neutrophils after neutrophils have adhered to activated endothelium^83^. Platelet–neutrophils interactions can also stimulate adhesion molecules and activate cytokine expression in neutrophils. Further activating platelets may stimulate entry of neutrophils into lesions and neutrophils that interact with platelets phagocytose bacteria more readily than unbound neutrophils. Furthermore, it has been shown that activated platelets can express P-selectin^24^ and E-selectin that are absolutely required for the delivery of neutrophils to the inflamed brain^84^. These generate the hypothesis that there is an interaction between platelets and neutrophils in relation to the risk of FS. Additionally, our study found a synergistic effect between MPR and NLR on an additive scale.

C-reactive protein (CRP), an acute-phase reactant secreted by the liver during inflammation, is considered one of the peaks of inflammatory markers. In this study, we found that CRP levels were significantly lower in children with FS compared to children without seizures and similar results were found by Romanowska *et al*.^28^. It can be suspected that children with FS develop inflammatory processes quickly enough that CRP levels do not reach their highest values. In children with fever without seizures, the inflammatory process was increasing slow enough to get CRP to higher levels^28,85^. Therefore, CRP is not identified as a factor affects the susceptibility of FS in this study.

We observed a statistically significant difference in NLR and MPV between the SFS group and the CFS group, whereas, no significant differences in the MPR, PLT and RDW values were observed between the two groups. To date, several studies have investigated the relationship between the inflammatory markers (NLR, PLT, MPV and RDW) and FS types, and suggested that NLR may be a practical predictor for differentiating FS types^29,31^. Consistently, in our study, NLR was significantly higher in the CFS group compared with the SFS group. Therefore, we believed that more inflammatory processes may have occurred in the brain of the patients with CFS. Particularly, ROC curve analysis of NLR in differentiating FS types was performed, and the optimal cut-off value of NLR was found to be 2.549. The sensitivity and specificity were 65.9% and 57.5%, respectively. The results are consistent with the previous studies^29,31^. Therefore, we agreed with the suggestion that NLR may provide clinicians with an insight into differentiating between simple and complex FS. Nevertheless, it is clear that the current sensitivity and specificity levels are moderate, far from assuring a definitive and objective differentiation.

The history for investigating MPV as a predictor for differentiating FS types is not long, but the evidence is increasing^27,29,31,32^. Nevertheless, the relationship between these factors and FS types was not consistent in different studies. For instance, Ozaydin *et al*.^32^, demonstrated that the MPV levels were significantly higher in the SFS group compared with the CFS group. However, Goksugur *et al*.^29^, and Yigit *et al*.^31^, revealed that there was no significant difference in MPV values between the two groups. Thus, the role of MPV in the differential diagnosis of two FS types is controversial. Although, we found that MPV was significantly higher in the SFS group than the CFS group in Chinese children, whether MPV will ever become a predictor for differentiating FS types remain to be seen. The physiological mechanisms of PLT and MPV in SFS and CFS will be explored first.

One limitation in our study should be addressed, because our study is a retrospective study, and in that serum cytokines such as IL-1β and TNF-α level were not measured normally in patients with FS. So there is no information on the levels of cytokines in our study. Experimental studies have concluded that IL-1β plays an important role in the pathogenesis of FS^46,50,51,86,87^. Several case-control studies also performed to measure the concentration of cytokines in the serum of seizure patients compared with that of healthy controls without seizures. However, conflicting results have been reported^88,89^. Choi *et al*. and Tutuncuoglu *et al*. reported that the serum IL-1β levels were significantly higher in FS patients than in the controls^3,42^. Haspolat *et al*.^65^ showed serum IL-1β and TNF-α level in patients with febrile seizures as compared to those in controls was not significantly different. Mahyar *et al*.^89^ showed serum IL-1β and TNF-α level in the simple and complex febrile seizure groups were significant lower than those in the control group. Another study also showed that the serum levels of the IFN-γ, IL-6, and IL-8 pro-inflammatory cytokines and the serum levels of the IL-10 and IL-1Ra anti-inflammatory cytokines were significantly higher in the FS children, however, serum IL-1β and TNF-α level in the two groups were not significantly different^88^. According above studies, IL-1β is an important factor influence the pathogenesis of FS. It does not mean any children with FS have higher level of IL-1β, we hypothesized that complex interactions among the above cytokines activation are involved in the pathogenesis of FS.

In conclusion, elevated NLR and MPR add evidence to the implication of white cells subsets in FS risk. Our results confirmed that NLR is an independent, albeit limited, predictor in differentiating between CFS and SFS. Moreover, NLR and MPR may have a synergistic effect that can influence the occurrence of FS.

## Data Availability

The datasets generated during the current study are available from the corresponding author on reasonable request.
